# Influence of Rapid Thermal Annealing (RTA) on the Properties of Indium Oxide Nanostructures

**DOI:** 10.3390/nano16090506

**Published:** 2026-04-23

**Authors:** Alina Matei, Cosmin Romanițan, Iuliana Mihalache, Oana Brîncoveanu, Vasilica Țucureanu

**Affiliations:** National Institute for Research and Development in Microtechnologies, IMT-Bucharest 126A, Erou Iancu Nicolae Str., 077190 Bucharest, Romania; cosmin.romanitan@imt.ro (C.R.); iuliana.mihalache@imt.ro (I.M.); oana.brincoveanu@imt.ro (O.B.)

**Keywords:** indium oxide, precipitation method, thin films, rapid thermal annealing (RTA)

## Abstract

In the present paper, In_2_O_3_ NPs were synthesized by a wet-chemical method, in the absence and presence of the surfactant, and deposited as thin films on silicon substrates. After deposition, the films were subjected to rapid thermal annealing (RTA) at 550 °C, 750 °C, and 900 °C, for 300 s, under an inert atmosphere. The correlation between the morphological, structural, and optical characteristics, the wetting capacity of In_2_O_3_ films synthesized under different synthesis conditions, and the influence of the RTA treatment are presented. The vibrations of In-O bonds for In_2_O_3_ samples were confirmed using FTIR spectroscopy. Structural analysis shows that In_2_O_3_ NPs have a cubic crystalline structure, but with the increase in temperature at 900 °C, diffraction peaks characteristic of the tetragonal phase of indium appear, correlated with a decrease in lattice parameters, as a result of the crystallinity. The morphology of the In_2_O_3_ samples was studied by SEM, revealing predominantly spherical and uniformly distributed particles with nanometric sizes. The absorption spectra of the In_2_O_3_ NPs showed peaks in the ultraviolet region, and the high energy bandgap value of the In_2_O_3_ films varied between 3.28 and 4.33 eV, depending on the samples and RTA treatment. The contact angle measurements of In_2_O_3_ films determined the wetting capacity of the surface, reflecting changes in surface morphology and structure induced by the RTA process. The results suggest that In_2_O_3_ thin films with spherical nanoparticles, good wettability, and percolation can be used for the development of sensors with increased selectivity and sensitivity.

## 1. Introduction

Advances in nanotechnology have led to the development of nanostructured oxide materials with unique properties (i.e., high surface-to-volume ratio, catalytic efficiency, reaction activity, sensitivity, selectivity, good adsorption capacity, modification to the desired size, etc.), enabling specific applications in different sectors to improve quality of life [1,2,3]. Among the intensively studied oxide materials, indium oxide (In_2_O_3_) nanostructures have started to gather particular interest due to their multifunctional properties, such as a direct energy bandgap in the range of 3.5 and 3.75 eV, an indirect energy bandgap of 2.62 eV, high transparency in the visible region, electrical conductivity, chemical stability, fast electron transfer capacity, low resistivity, and high mobility. Also, this oxide is a transparent conducting semiconductor that usually crystallizes in the cubic bixbyite-type phase (called c-type rare-earth oxide structure), considered a good insulator in the stoichiometric state, which becomes highly conductive in its non-stoichiometric In_2_O_3−x_ form due to oxygen deficiency and the increased number of defects (intrinsic or native) acting as donors to generate free electrons. In addition, it has gained considerable attention in a wide range of technological applications, including optoelectronic devices, gas sensors, solar cells, electrochemical biosensors, field-emission transistors (FET), light-emitting diodes (LEDs), transparent windows in liquid crystal displays, flat panel displays, photocatalysis, supercapacitors, etc. [4,5,6,7,8,9].

Currently, numerous deposition processes are known to synthesize In_2_O_3_ nanostructures with different dimensions and morphologies (i.e., nanoparticles, nanowires, nanotubes, etc.), ensuring a high control over the physicochemical properties. Until now, the most well-known processes are thermal decomposition, physical vapor deposition, laser ablation, electrochemical deposition, electron beam evaporation, RF magnetron sputtering, atomic layer deposition, sol–gel processes, (co)precipitation, hydrothermal, spray pyrolysis, sonochemical, or vacuum evaporation, each of which is being developed to fulfill the targeted applications [9,10,11,12]. The morphological, structural, compositional, optical, and electrical properties of In_2_O_3_ nanostructures are influenced by the synthesis method and experimental conditions (i.e., precursor types, solution concentration, substrate type, deposition rate, post-deposition annealing temperature, gas flow, film thickness etc.). Usually, different deposition methods such as chemical vapor deposition, spray pyrolysis, indium evaporation followed by oxidation, high vacuum thermal evaporation, and RF/DC magnetron sputtering are intensively used for preparing transparent and conducting films of In_2_O_3_ [13,14,15,16].

Research shows that for the formation of In_2_O_3_ films on different substrates (SiO_2_/Si, quartz, glass, ITO, sapphire, Al_2_O_3_, FTO, etc.) it is necessary to fulfill certain process conditions during deposition, as well as the post-growth conditions performed after the growth of the films. The main drawbacks of the known processes are related to relatively high thermal treatment temperature (800–1500 °C), long holding time, and controlled atmosphere conditions (nitrogen, argon and oxygen), which are important characteristics for improving the quality and performance of the material. In_2_O_3_ thin films with high crystalline quality and favorable photoelectrical characteristics can be obtained depending on the deposition conditions, morphology, and substrate type. These films exhibit high average transparencies in the visible region (83–90%) and wide optical bandgaps (3.62–3.75 eV), excellent chemical/physical properties, making them promising candidates for detection of gasses (e.g., etanol, ozon, H_2_S, acetone, NO_x_, chlorine, ammonia, VOCs), platforms for efficient light-assisted oxidation of water and reduction of CO_x_, and biosensor arrays based on field-effect transistors to detect dopamine, glucose in body fluids, exosomal circulating microRNA with peptide nucleic acid probes, etc. [17,18,19,20,21,22,23,24,25,26].

Depending on the targeted structures and morphologies of In_2_O_3_, the solution-based route offers an alternative for large-area, high-throughput deposition of thin films, enabling easy composition control and low-cost processing. Moreover, chemical synthesis protocols offer the advantage of improving the quality of In_2_O_3_ nanostructures by simultaneously monitoring synthesis parameters, such as the type and concentration of precursors, amount of solvent, temperature and reaction time, and presence/absence of surfactants, with the thermal treatment procedures. A wide variety of precursors (e.g., indium nitrates, chlorides, acetates, sulfates, etc.), reaction media (e.g., water, ethanol, isopropanol, etc.), various surfactants/tensioactive agents (i.e., CTAB, SDS, SDBS, Triton X-100, etc.), and chelating agents (e.g., citric acid, acetylacetone, etc.) were used, as these accelerated the growth of preferential orientation and ensured good control of In_2_O_3_ nanostructures with different structures and morphologies, such as nanoparticles, wires, rods, plates, fibers etc. [27,28,29,30,31].

The synthesis of In_2_O_3_ by the wet-chemical method presents the advantage of process simplicity, low acquisition costs, the ability to adjust nucleation and agglomeration tendency, control of particle size and morphology, and optimization by tuning the synthesis parameters (i.e., precursor concentrations, reaction temperature, duration, atmospheres, etc.). Usually, films obtained by chemical methods require thermal treatments to remove impurities from the precursors used, improve the structural characteristics, and stabilize the oxide surface [1,32,33,34].

In this context, the thermal treatment conditions (e.g., time and temperature of sintering, gas atmosphere) play a critical role in determining the specific crystalline phases of In_2_O_3_. Furthermore, these treatments significantly affect the functional properties of the synthesized materials. Among them, rapid thermal annealing (RTA) has gained considerable attention owing to its advantages, such as rapid heating to high temperatures (within minutes or less), lower energy consumption, vacuum conditions or inert atmospheres, uniform gas distribution, and controlled cooling to ambient temperature, etc. Compared with conventional thermal treatments, RTA ensures improved process reproducibility, enhanced thin film densification, superior properties, and better uniformity. Numerous studies have reported the use of RTA for the treatment of a wide range of materials with various applications, including WO_3_ thin films for energy-efficient smart windows, CuO/Cu_2_O films used in solar cells and photonic devices, ZnO- and IGZO-based thin-film transistors and high-sensitive devices, and ITO for the fabrication of transparent contact electrodes in solar cells, etc. [35,36,37,38,39,40,41,42,43,44,45].

In this paper, a chemical method for the synthesis of oxide precursors, their deposition as thin layers, and thickness control by the number of layers deposited on a silicon substrate were investigated. Furthermore, we report the optimum annealing conditions for the preparation of high-quality In_2_O_3_ films by applying thermal treatments at different temperatures (at 550 °C, 750 °C and 900 °C, for 300 s), for a short duration, in an inert gas atmosphere, enabling efficient and precise process control. The novelty of this work lies in the application of rapid thermal annealing (RTA) for the synthesis of In_2_O_3_ films, through systematic control of the sintering variables (time, temperature, and atmosphere) to establish optimal conditions, and evaluation of their effects on the structural defects of films and crystallinity improvement. In addition to the treatment conditions, this study explores the possibility of improving the morpho-structural properties, and hydrophilic character of the nanostructured surface to enhance the technological performance for advanced biosensors based on field-effect transistors. The main advantages of the proposed precipitation method include a low risk of contamination, reduced costs, and precise control over the synthesis parameters, etc.

## 2. Materials and Methods

### 2.1. Materials Used

All the chemical reagents, indium (III) nitrate [In(NO_3_)_3_ xH_2_O], ammonium hydroxide [NH_4_OH], anionic surfactant sodium dodecyl sulfate [C_12_H_25_NaO_4_S, SDS], ethanol [C_2_H_6_O], acetone [C_3_H_6_O], isopropyl alcohol [C_3_H_8_O, IPA], deionized water [H_2_O, DWA], sulfuric acid [H_2_SO_4_] with hydrogen peroxide [H_2_O_2_], used in our research are of analytical grade (provided by Carl Roth and Merck) and without further purification. Also, we used a silicon wafer as the testing substrate. To remove any contaminants from the surface of the silicon substrates (1.5 cm × 1.5 cm) the standard procedure for cleaning the wafers is used, which involves: firstly, the substrates were cleaned with C_3_H_6_O and C_3_H_8_O, and dried with nitrogen gas flow, followed by treatment with Piranha solution (H_2_SO_4_:H_2_O_2_).

### 2.2. Synthesis Process

The precipitation method was used to synthesize In_2_O_3_ nanoparticles in the absence and presence of a surfactant. The aqueous stock solutions of In(NO_3_)_3_ [10 mM], SDS [1 mM], and NH_4_OH [10 mM] were freshly prepared just before use. The nitrate solutions were transferred into laboratory glasses, and, for the surfactant-assisted samples, the SDS solution was added to the In^2+^ salt solution. Then, both solutions were heated at 70–80 °C, under continuous stirring. Over these solutions, under continuous stirring, the ammonia solution was added at a rate of approximately 0.1 mL/s, until the pH of the solution reached and maintained a value of 9. The stirring was continued, at about 80 °C, for 3 h. The formed colloidal solutions were allowed to stand overnight for maturation and precipitation throughout the mass. The supernatant was removed by centrifugation and decantation, and the obtained precipitates were washed with a mixture of water and ethyl alcohol. The centrifugation, settling, and washing steps were repeated 3 times to remove secondary reaction products. The process to obtain In_2_O_3_ without surfactants involves removing SDS from the technological flow.

### 2.3. Deposition of the Oxide Precursor as Thin Films

The oxide precursors were deposited using the “spin coating” method as thin layers on previously cleaned silicon substrates according to standard technology, at a rotational speed of about 2500 rot/min. The coated substrates were vacuum-dried at room temperature for 12 h, prior to RTA treatment.

### 2.4. Rapid Thermal Annealing (RTA)

**Figure 1:**
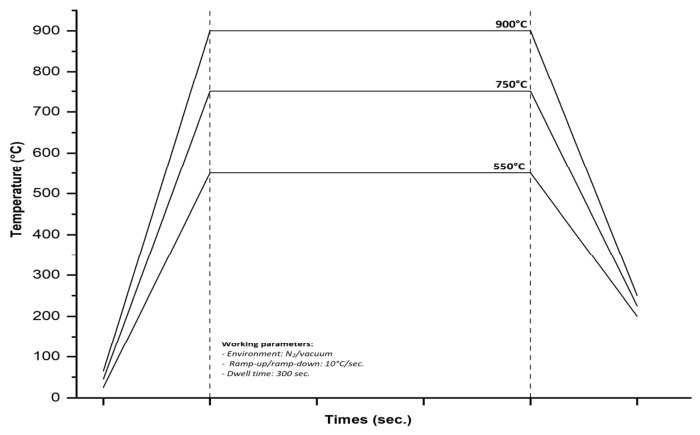
Representative diagram of RTA treatment highlighting process conditions.

For the thermal treatment, a rapid thermal processing furnace (AS-One 100) from Annealsys (Montpellier, France) was used, which involves heating the substrates to a high temperature (~1250 °C), with a ramp rate of 0.1–200 °C/s, vacuum of up to 10^−6^ Torr, in inert gas (nitrogen and argon) for short periods using high-intensity lamps, followed by controlled cooling of the samples. Rapid thermal annealing (RTA) treatments were carried out in the stainless-steel cold-wall process chamber of the equipment, where the samples were positioned between a silicon carbide-coated graphite susceptor and a cover. Heating was provided by the halogen lamps installed on the upper side of the process chamber, allowing a good temperature uniformity, and the thermal transfer between the tubular lamp and substrates. During the process, nitrogen gas was introduced into the chamber, and the working pressure was maintained at about 3.24 × 10^−2^ mbar. The samples were heated at different annealing temperatures (in the range of 550–900 °C), with a ramp rate of about 10 °C/s and dwell time of 300 s. After annealing, the furnace was cooled with the same ramp rate (10 °C/s) and maintained under the vacuum until a temperature below 200 °C was reached (Figure 1).

### 2.5. Processing and Characterization Equipment

Structural, morphological, optical, and wetting capacity analyses of the synthesized samples were performed using the following equipment:

Fourier transform infrared spectrometry (FTIR) was used to study the configurations of chemical bonds in oxide precursors and oxide samples obtained from treatments at temperatures of 550–900 °C. A Tensor 27 spectrometer (Bruker Optics, Karlsruhe, Germany) equipped with an ATR Platinum accessory with a single-reflection diamond crystal was used. All samples were drawn after 64 scans, with a resolution of 4 cm^−1^, over a spectral range of 4000–370 cm^−1^. The spectra were acquired and processed using the OPUS software (version 6.0).

X-ray diffraction (XRD) analysis was used to determine the crystalline structure, crystallite size, and purity of the In_2_O_3_ thin films, employing CuKα radiation (λ = 1.5406 Å), with an operating voltage of 45 KV and a current of 100 mA. All XRD patterns were acquired in the 2θ range of 10–80° at room temperature with a scanning speed of 6°/min and a minimum step of 0.01°/min.

The surface morphological studies of as-deposited and annealed films are accomplished by using a FEI Nova NanoSEM 630 scanning electron microscope (FEI Company, Hillsboro, OR, USA) equipped with a through-the-lens detector (TLD) at a magnification varying between 50 and 100 kx, at an acceleration voltage of 10 kV. Energy-dispersive X-ray (EDX) analysis at an acceleration voltage of 15 kV (Smart Insight AMETEK, Inc., Berwyn, PA, USA) was used to characterize the elemental composition.

The size distribution of the In_2_O_3_ nanoparticles was obtained from SEM images by measuring around 200 nanoparticles. The program “Image J” (software Version IJ 1.46r) was used to extract the necessary data and to plot the histogram. By the application of the Gaussian function, the average NP size and the standard deviation were determined.

The diffuse reflectance spectra (DRS) of the samples were recorded using an integrating sphere mounted inside a Cary 5000 spectrophotometer (Agilent Technology, Santa Clara, CA, USA), from 200 to 700 nm at a resolution of 1.0 nm. All samples were baseline corrected using the PTFE standard. Further, the Kubelka–Munk transformation F(R) was employed to determine the optical band gap energy (E_g_).

Contact angle (CA) measurements were performed to investigate the surface wettability capacity of In_2_O_3_ thin films, using a Theta Optical Tensiometer (KSV Instruments, Helsinki, Finland) equipped with the CAM 101 camera and a 1394 firewire interface for fast image acquisition. We used water as the testing liquid, and droplets with a volume varying between 1.5 and 2 μL were deposited on the surface of samples. The measurements were made at least three times, and the reported value was their average.

## 3. Results

### 3.1. Functional GROUP Analysis (FTIR)

**Figure 2:**
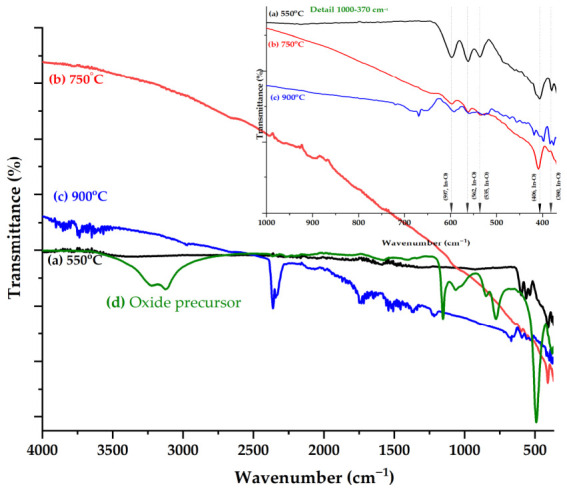
ATR-FTIR spectra of the oxide precursor In_2_O_3_ films obtained after treatment at different RTA temperatures (550 °C, 750 °C and 900 °C).

Indium oxide (In_2_O_3_) is a polymorphic compound, consisting of five atoms that can be attributed to the C_2v_ symmetry group, for which, theoretically there can be nine vibration modes of the molecule. The most representative FTIR spectra of the oxide precursor and In_2_O_3_ samples treated using RTA at 550 °C, 750 °C, and 900 °C are shown in Figure 2. The four polymorphic forms of oxide have similar vibrational structures, with a slight variation in wavenumber or intensity determined by In^3+^ distribution in the interstitial sites or the appearance of some defects [46].

Bands associated with the ring mode, torsion, and out-of-plane bending vibrations can be observed. For the sample treated at 550 °C, the formation of the cubic phase of In_2_O_3_ was confirmed as a result of the appearance of bands below the 600 cm^−1^ bands that can be attributed to the symmetrical and asymmetrical vibration modes of the In-O bonds (597, 562, 536 cm^−1^ (υ_as_(In-O), B_2_), 406 and 380 cm^−1^ (υ_s_(In-O), A_1_)). Also, no bands were observed that could suggest the existence of compounds that remained undecomposed. As in the case of samples treated at 550 °C, those treated at 750 °C are characterized by bands that can be associated with the vibrations of bonds in the crystal lattice of In-O, bands observed at 599, 564, 536 cm^−1^ (υ_as_(In-O), B_2_), 411 and 384 cm^−1^ (υ_s_(In-O), A_1_), confirming the existence of the same type of crystal structure [47].

FTIR spectroscopy further supported the XRD observations for samples annealed at 900 °C. Simultaneously with the slight displacement of the bands observed in the case of the previous samples (592, 559, 527, and 418 cm^−1^) the appearance of bands (669, 658, 472, 456, and 398 cm^−1^) was observed, confirming the change in the crystallinity of the oxide. It was also observed that the peaks around 1550 cm^−1^ became sharper as the thermal treatment temperature rose to 900 °C [1,48].

For all spectra, bands in the spectral range of 4000–1200 cm^−1^ can be associated with water molecules adsorbed during sample handling.

### 3.2. Structural Analysis (XRD)

The information obtained by FTIR spectroscopy regarding the occurrence of variations in the spectra of samples treated at high temperatures highlighted the need for further studies; for this reason, the XRD technique was used to evaluate the formed crystalline phases. XRD analysis was used to identify the crystal structure, mean crystallite size, unit cell parameters, lattice volume, and lattice strain of oxide samples obtained from precursors with and without SDS surfactant, and subjected to RTA treatment at 550, 750, and 900 °C.

**Figure 3:**
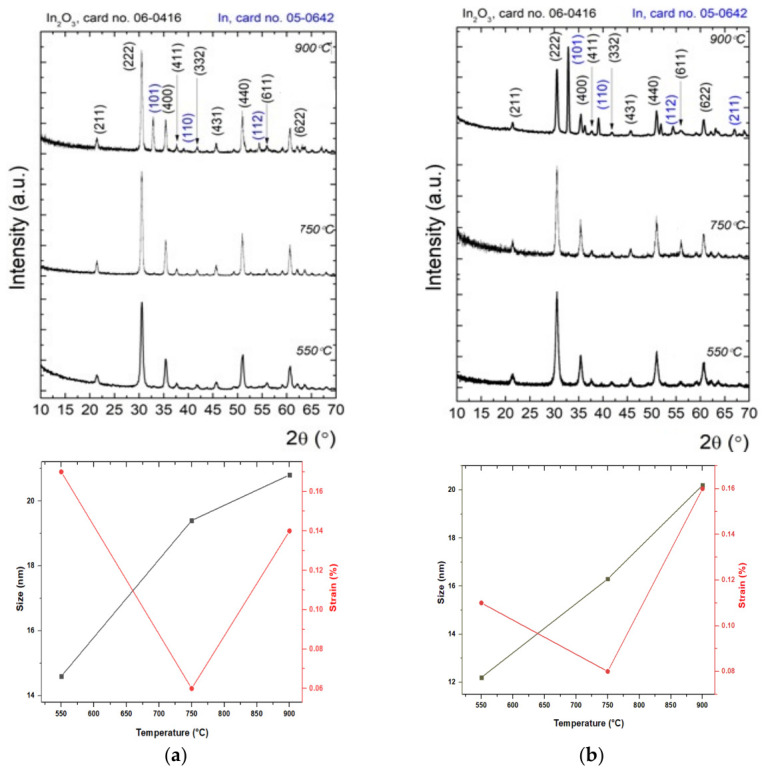
XRD patterns and relationship of crystallite size and strain of In_2_O_3_ films obtained in the absence (**a**) and presence of surfactant (**b**) as the function of RTA temperatures (550 °C, 750 °C and 900 °C).

Figure 3a,b show the evolution of XRD models of In_2_O_3_ samples in the absence and presence of surfactant, after thermal treatments at different temperatures. The diffraction lines for the In_2_O_3_ samples have been indexed according to the International Centre for Diffraction Data (ICDD) database with cards no. 06-0416 (In_2_O_3_) and 05-0642 (In), and Table 1 presents the mean crystallite size (D), lattice strain (ε) unit cell parameters (a = b = c), and unit cell volume (V). For In_2_O_3_ samples treated at 550 and 750 °C (regardless of their type, with or without surfactant), there appear to be intense peaks at 2θ = 21°, 30°, 35°, 37°, 41°, 45°, 51°, 55° and 60° assigned to the (211), (222), (400), (411), (332), (431), (440), (611), (622) planes of In_2_O_3_, respectively. One can observe that the diffraction lines are in good agreement with the standard JCPDS cards no. 06-0416 and perfectly indexed with the cubic crystalline structure of indium oxide. At the higher temperature of 900 °C, the metallic indium (In) and In_2_O_3_ co-exist, as shown in the XRD pattern. Also, the diffraction peaks at 2θ = 30°, 39°, and 54° are assigned to the (101), (110), and (112) planes, which correspond to the metallic indium with the tetragonal structure, being in accordance with JCPDS card no. 05-0642 [49,50,51].

The crystal quality was evaluated by the Rietveld model, in which the experimental data were described by a theoretical model that encodes information about the crystal system (e.g., unit cell parameters, lattice strain, mean crystallite size) [52,53,54]. According to the data presented in Table 1, it can be seen that regardless of the type of In_2_O_3_ sample in the absence or presence of surfactant, the only differences occur in samples heat-treated at high temperature up to 900 °C. Thus, the relationships of crystallite size, strain and RTA temperature are shown in Figure 3a, where the mean crystallite size increased monotonically from 16 nm (at the temperature of 550 °C) to 23.9 nm (at the temperature of 750 °C), reaching 27.1 nm (at the temperature of 900 °C). For samples of In_2_O_3_ in the presence of surfactant (Figure 3b), during temperature increase, the average crystallite size increases from 13.8 to 18.5 nm and then to 24.8 nm. It is found that the increase in mean crystallite size with the temperature of the RTA thermal treatment, and the use of surfactant, leads to a relative change in the intensity of various diffraction peaks. According to the presented observations, the treatment temperature and the crystallite size are directly correlated; it is found that high temperatures improve crystallinity and increase the average crystallite size due to accelerated grain growth and diffusion.

Furthermore, the microstrains of In_2_O_3_ NPs for different RTA temperatures (550 °C, 750 °C, and 900 °C) were varied from 0.05 to 0.17%. The results obtained indicate that strain is dependent on crystallite size, due to the high density of grain boundaries and dislocations. Based on the obtained results, it can be inferred that the temperature of 550 °C applied to In_2_O_3_ NPs led to the smallest crystallite size for both types of samples. In the case of RTA treatments at temperatures of 550 °C and 750 °C, in addition to the slight displacement of the tips, an increase in the lattice parameters is observed, compared to samples without surfactants. And, with the increase in the temperature to 900 °C, there is a decrease in the height of the vertices characteristic of the cubic structure of In_2_O_3_ and the additional appearance of peaks characteristic of the tetragonal phase of indium, correlated with a slight decrease in parameters, as a result of the decrease in crystallinity. By comparing the results, an increase in the average size of the crystallites, the improvement of surface morphology of films, and their uniformity depending on the temperature of the thermal treatment is observed. These modifications can be attributed to the increase in the density of nucleation and the mobility of electrons. Thus, the use of surfactant and the increase in temperature induces an improvement in the quality of the crystal in the samples [55,56,57].

### 3.3. Morphological and Compositional Analysis

The size, shape, and agglomeration tendency, respectively, and elemental composition of the In_2_O_3_ nanoparticles were analyzed by scanning electron microscopy (SEM), equipped with an energy-dispersive X-ray (EDX) spectroscopy system.

**Figure 4:**
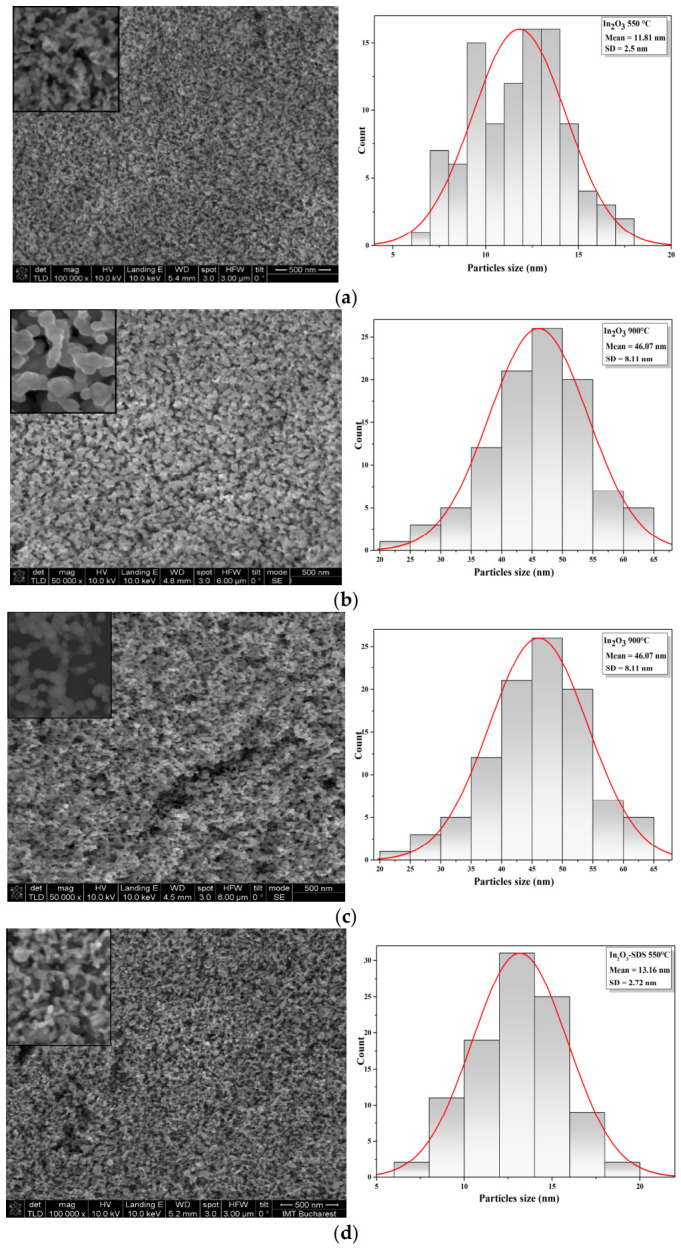

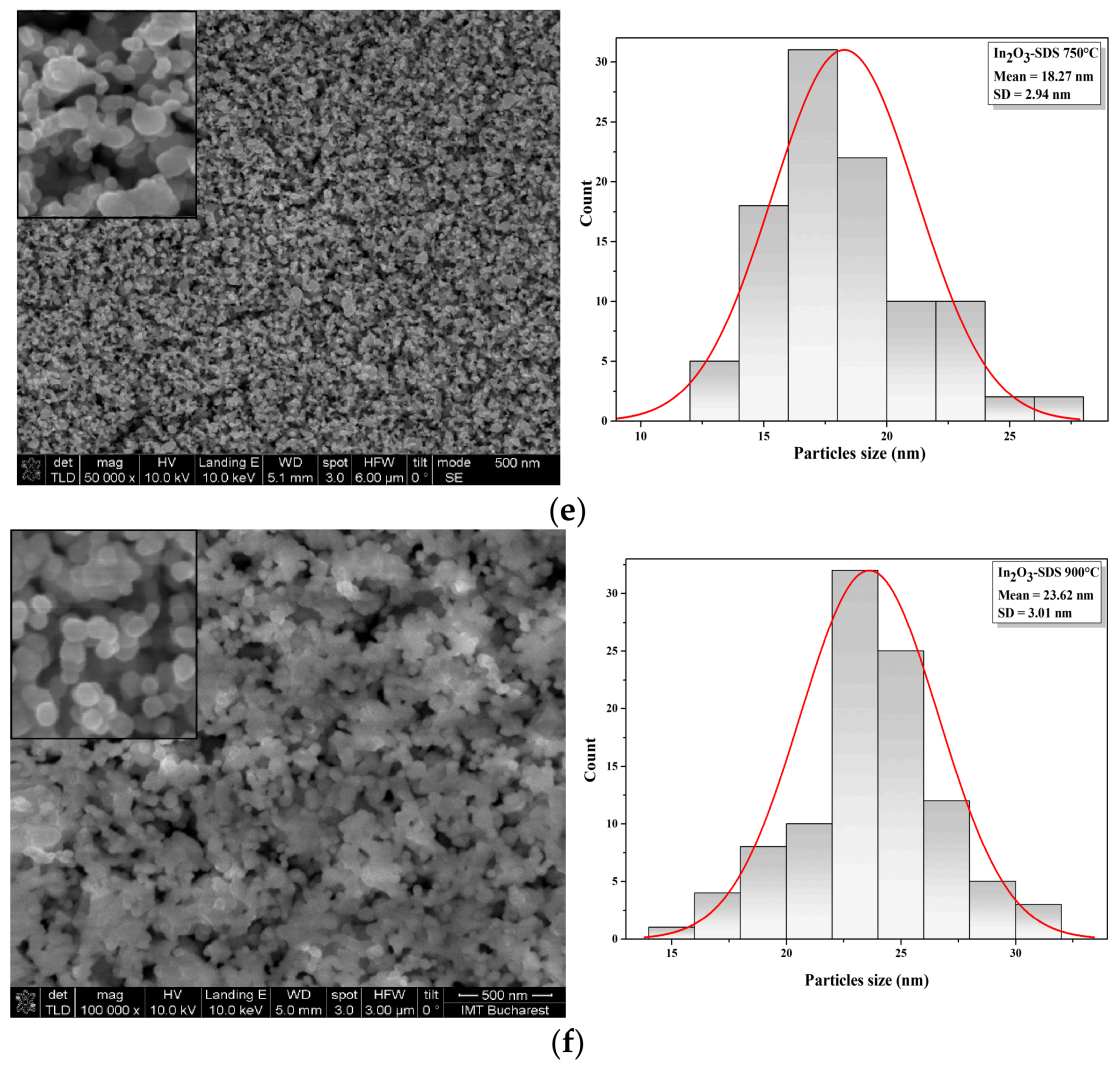
SEM micrographs and frequency histogram of the In_2_O_3_ films obtained in the absence (**a**–**c**) and presence of surfactant (**d**–**f**) and treated at different RTA temperatures (550 °C, 750 °C and 900 °C).

The morphological analysis and the histograms of the In_2_O_3_ samples in the absence and presence of surfactant, after thermal treatments under different temperature conditions, is presented in Figure 4a–f. SEM images of the In_2_O_3_ samples synthesized in the absence of surfactant (Figure 4a–c) reveal that the overall morphology does not change significantly with annealing temperature. The particles have irregular shapes and small sizes, which determines an agglomeration tendency. After rapid thermal treatment at higher temperature (750 °C and 900 °C), the SEM micrographs indicate the formation of uniform, homogeneous and dense surfaces. It also demonstrates the presence of agglomerations on the surface of the oxide film, compared to those treated at 550 °C. The histogram curves showed that the average size of the synthesized nanoparticles of In_2_O_3_ without surfactant varied in the range of 5–17 nm, 15–36 nm and 23–62 nm, depending on the applied thermal treatment. The morphological analysis of the In_2_O_3_ samples in the presence of surfactant (Figure 4d–f) indicates the formation of predominantly spherical particles, delimited from each other, with relatively uniform distribution, with nanometric particle sizes. In addition, the obtained In_2_O_3_ nanoparticle size histograms showed sizes ranging from 7 to 30 nm, with mean diameter 13.6 ± 2.72 nm, 18.27 ± 2.93 nm, and 23.62 ± 3 nm (±standard deviation), variations that depend on the treatment applied to the samples. In the case of In_2_O_3_ samples (in the absence and presence of surfactant) thermal-treated at a temperature of 550 °C, the nanoparticles exhibit a uniform morphology and very small sizes. Increasing the RTA temperature to 750 °C and 900 °C, leads to partial fusion of the granules, resulting in larger particles, with sizes reaching up to 60 nm, while maintaining their spherical morphology. It has been found that surfactants may be used to reduce interfacial energy and control particle growth mechanisms by forming an absorption layer on the particle surface, thereby influencing morphology, size, and agglomeration tendencies [58,59,60].

**Figure 5:**
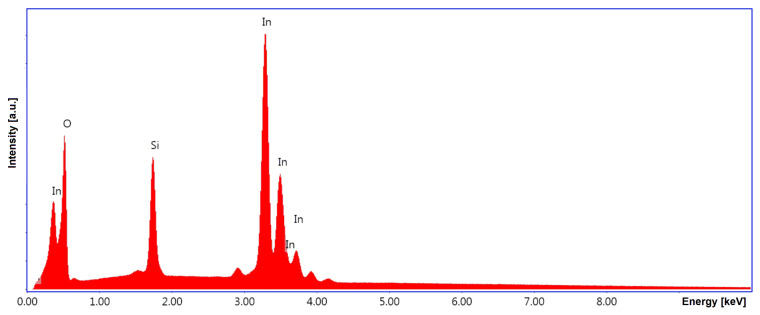
The EDX spectrum for a representative In_2_O_3_ film deposited on SiO_2_/Si following an RTA treatment.

To evaluate the chemical composition at the atomic level, Figure 5 shows the EDX spectrum for a representative sample of In_2_O_3_ deposited on an SiO_2_/Si substrate and treated by RTA treatment. From a qualitative analysis perspective, peaks were identified in the sample spectrum that can be attributed to oxygen atoms originating from both the In_2_O_3_ film and the substrate (O(K) at 0.52 keV), as well as to indium atoms (In(L) with multiple peaks in the 3–4 keV range (with the dominant peak characteristic of In(L_α_) at 3.29 keV and a secondary peak at In(L_β_) at 3.49 keV) and In(M) at 0.37 keV), and silicon due to the substrate (Si(K) at 1.74 keV) [61].

### 3.4. Optical Analysis

**Figure 6:**
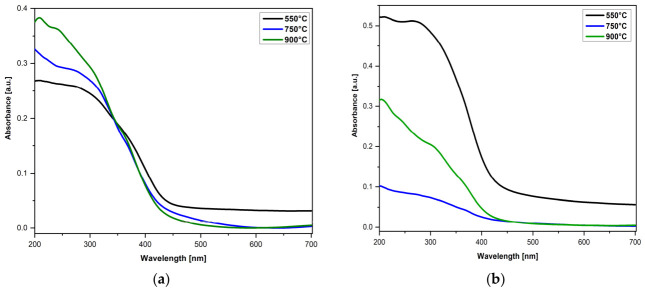
The DRS absorbance of In_2_O_3_ films obtained in the absence of surfactant (**a**) and in the presence of surfactant (**b**), and treated at different RTA temperatures (550 °C, 750 °C and 900 °C).

The diffuse reflectance spectroscopy (DRS) absorption spectra of In_2_O_3_ nanoparticles in the absence and presence of a surfactant, after heat treatments at different temperatures, were recorded in the range of 200–700 nm and are illustrated in Figure 6. The absorbance of the In_2_O_3_ films is influenced by both the synthesis conditions (in the absence and presence of surfactants), and the temperature of the RTA treatment. As can be seen, the spectra of the In_2_O_3_ samples show absorption bands in the ultraviolet region, which correspond to the direct transition from the valence band to the conduction band, occurring when the photon energy exceeds the band gap energy of the semiconductor [62].

In the case of In_2_O_3_ samples, regardless of their type (Figure 6a,b), a variation in absorbance was observed, which depends on the applied RTA temperature. The synthesized samples show different absorption spectra due to the presence of different oxygen free sites between the particles and possibly due to the quantum confinement effect. The mechanism underlying the enhanced absorbency may be related to the effect of increasing surface roughness and the size distribution of the particles present in the films. In addition, in the case of In_2_O_3_ samples in the presence of surfactant (Figure 6b) it is observed that the intensity of the band for samples treated at a temperature of 550 °C is relatively high, due to low particle size and the excitonic transition of the valence band electrons to the conduction band, respectively [63,64].

**Figure 7:**
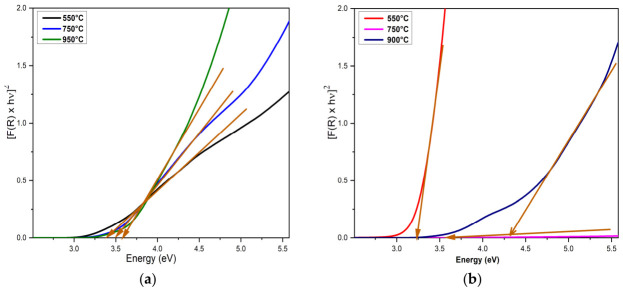
The energy band gap of In_2_O_3_ films obtained in the absence of surfactant (**a**) and in the presence of surfactant (**b**), and treated at different RTA temperatures (550 °C, 750 °C and 900 °C).

The values of the direct energy band gap of In_2_O_3_ films of different RTA temperatures were determined using the Kubelka–Munk transformation and are graphically presented in Figure 7. The absorption coefficient of the samples was calculated using the K-M function from diffuse reflectance spectra, according to the equation:

$${F\left(R\right)=\frac{\left(1-R\right)}{2R}}^{2}$$

where F(R) is the K-M function, and R indicates the diffuse reflectance [65,66].

Also, the band gap is obtained using the equation:

(*F*(*R*) × *hν*)^2^ = *A*(*hν* − *E_g_*)

where hν is the incident photon energy, A is a proportional constant, and E_g_ is the optical band gap.

The results showed that the bandgap energy of the In_2_O_3_ samples varies depending on both the absence/presence of the surfactant and the temperature of the RTA treatment applied. Thus, the calculated band energies (E_g_) increased slightly from 3.28 eV for the In_2_O_3_ sample treated at 550 °C, to 3.41 eV for the In_2_O_3_ sample treated at 750 °C, to 3.51 eV for the In_2_O_3_ sample treated at 900 °C. The results showed that increasing RTA temperature from 550 to 750 and 900 °C increases the bandgap energy of the In_2_O_3_ samples in the presence of the surfactant from 3.63 eV, to 3.87 eV and 4.33 eV, respectively. This variation in the band gap, from 3.28 eV to 4.33 eV, can be attributed to both the presence of surfactant during synthesis and the RTA treatment temperature. Surfactants significantly influence the band gap of In_2_O_3_ nanoparticles by modifying nucleation and growth kinetics, affecting the size, morphology, crystalline structure, and surface defects of the nanoparticles. Also, the heat treatment is a determining factor, because with increasing temperature there is a modification of the band gap energies, which can be attributed to the increase in grain size, the loss of electron energy of the incident beam on the surface, and the reduction in disorder, as well as the improvement of the film quality, probably due to a lower defect density. The obtained energy bandgap values (Table 2) corresponding to the thin films with the In_2_O_3_ crystalline phase are in good agreement with other reports in the literature [59,67,68,69].

### 3.5. Wetting Capacity

The wetting capacity was evaluated by measuring the contact angle, which describes the interaction between the liquid droplet with the solid surface. Depending on the value of the angle, the hydrophobic character is determined, with an angle greater than 90°, where the liquid forms a compact droplet of liquid, indicating low wettability, adhesion, and surface free energy. At the same time, a small contact angle of 90° suggests a high wettability, which means the liquid spreads easily across the surface. Based on the above results, the contact angles of the In_2_O_3_ films are greater than 90°, which suggests that the films exhibit changes in the direction of high hydrophobicity [70,71,72].

**Figure 8:**
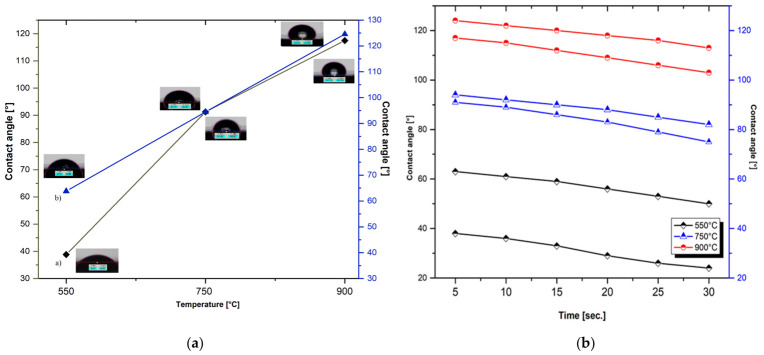
The contact angle (**a**) and evolution of the retention time of the water droplet (**b**) on the surface of In_2_O_3_ films obtained in the absence and presence of surfactant, and treated at different RTA temperatures (550 °C, 750 °C and 900 °C).

The contact angles (CA) between a liquid droplet and the In_2_O_3_ film surface were measured as a function of the RTA treatment at different temperatures, and the corresponding results are presented in Figure 8a.

To determine the average value of the angle, three measurements were performed for each sample, with the reported value representing the mean of the left and right angles formed between the solid surface and the tangent to the droplet profile at the intersection point. For the measurements, deionized water was used as a reference liquid, with a controlled droplet volume of ~2 μL. Based on the values of the contact angles of the In_2_O_3_ samples in the absence or presence of the surfactant, thermal-treated at a temperature of 550 °C, it is found that the angles vary from 38° to 63°, which suggests that the films show changes in the direction of maintaining the same hydrophilic behavior. The high degree of wetting can be associated with increased roughness of RTA-treated In_2_O_3_ films at relatively low temperatures. By applying the RTA treatment at high temperatures, it is observed that the values of the contact angles vary significantly and the character changes from hydrophilic to hydrophobic. This increase in angles may be due to the surface tension that decreases when the temperature increases, the results being in good accordance with the literature [73].

The percolation capacity is quantified by the variation in the contact angle as a function of water absorption time on the oxide film surface, and the percolation rate is the speed at which this occurs [74]. According to the graphic representation in Figure 8b, a slight decrease in the angle value is observed with the increase in the holding time, by losing the hydrophilic character and increasing the hydrophobicity of the samples treated at temperatures above 750 °C. The results obtained are largely in agreement with the observations of the SEM analyses; the increase in roughness influences the surface properties favoring the increase in the contact angle of the water on the surface of the thermal-treated In_2_O_3_ films (in the absence and presence of the surfactant).

## 4. Conclusions

In_2_O_3_ films were synthesized by the precipitation method, in the absence and in the presence of a surfactant. The resulting oxide precursors were deposited as thin films on silicon substrates, and subsequently annealed at 550 °C, 750 °C and 900 °C under an inert atmosphere, using a heating ramp rate of 10 °C/min, and a dwell time of 300 s. The influence of the RTA treatment on the properties of the In_2_O_3_ films was investigated in detail. All samples were investigated in terms of morphology, structure, and optical properties of In_2_O_3_ as well as wetting capacity. The study was carried out over a temperature range from 400 °C to 1000 °C, with the aim of determining the optimal process temperature. The three selected temperatures were chosen for their relevance to structural evolution; thus, the minimum temperature of 550 °C can be associated with the transition from the amorphous to the crystalline phase, while at the maximum temperature of 900 °C, the coexistence of two phases (In_2_O_3_ and metallic In) is observed for the first time.

The FTIR spectra of the In_2_O_3_ samples treated at 550 °C and 750 °C exhibit characteristic absorption bands in the 600–380 cm^−1^ range, corresponding to the symmetrical and asymmetrical vibrational modes of In-O bonds. With the increase in temperature to 900 °C, a slight displacement of the bands and an increase in the intensity of these peaks confirm the changes in crystallinity of the oxide following high-temperature treatment. XRD analysis revealed that the mean crystallite size and lattice parameters of the In_2_O_3_ NPs increased as a function of the RTA temperature, highlighting an enhancement of crystallite growth from ~13 to 25 nm, at high temperatures. Furthermore, the SEM images confirmed the formation of predominantly spherical particles with nanometric dimensions for all In_2_O_3_ samples. At a high temperature of 900 °C, the nanoparticles tend to agglomerate and increase the particle size up to 60 nm, while keeping the spherical morphology. These experimental results indicate that the applied thermal treatment parameters can be considered critical factors in the formation of In_2_O_3_ NPs with different shapes and sizes, relatively uniform distribution and reduced agglomeration tendency. The histograms of the synthesized In_2_O_3_ samples indicate a variation in the average particle diameter, which depends on the applied thermal treatment. In addition, EDX analysis confirmed the presence of indium and oxygen as the main elements, with no significant impurities.

The DRS absorption spectra show that the bandgap energy value varies depending on the sample types synthesized in the absence and presence of surfactants, and the temperature of RTA heat treatment. Also, all the In_2_O_3_ samples show a significant increment of the absorption edge in the ultraviolet region. The analysis of the contact angle with water and the evolution of the retention time of the water droplet on the surface of In_2_O_3_ films in the absence and the presence of surfactant shows a transition from hydrophilic to hydrophobic behavior at high temperatures.

Based on the obtained results, In_2_O_3_ exhibits special behavior under different synthesis conditions and thermal treatments, exhibiting distinct properties, and excellent performance, which highlights its potential for advanced applications.

## Figures and Tables

**Table 1:** Mean crystallite sizes, lattice parameters and atomic planes indexed with Miller indices for In_2_O_3_ samples with thermal treatment at different RTA temperatures.

Diffraction Parameters	In_2_O_3_ in the Absence of Surfactant			In_2_O_3_ in the Presence of Surfactant		
	550 °C	750 °C	900 °C	550 °C	750 °C	900 °C
Mean crystallite size [nm] Strain [%]	14.6	19.4	20.8	12.2	16.3	20.2
	0.17	0.057	0.14	0.108	0.08	0.16
Lattice parameters						
a = b = c [Å]	10.12	10.124	10.118	10.118	10.126	10.12
Atomic cell volume [Å^3^]	1035.81	1038.43	1036.40	1036.09	1037.66	1035.81
χ^2^	~1	~1	~1	~1	~1	~1
Phase (Miller indices)			2θ			
(201)	21.42	21.46	21.47	21.46	21.34	21.42
(222)	30.51	30.52	30.53	30.49	30.52	30.53
(101)	-	-	32.83	-	-	32.86
(400)	35.39	35.39	35.43	35.44	35.39	35.41
(411)	37.59	37.63	37.68	37.57	37.59	37.68
(110)	-	-	39.06	-	-	39.06
(332)	41.68	41.74	41.74	41.79	41.69	41.67
(431)	45.68	45.6	45.61	45.55	45.59	45.65
(521)	49.36	49.29	49.17	49.15	49.16	49.33
(440)	50.96	50.96	50.86	51.03	50.96	50.97
(112)	-	-	54. 4	-	-	54.24
(611)	55.9	55.93	56.02	55.95	56.13	56.03
(622)	60.56	60.61	60.64	60.58	60.57	60.6
(631)	62.15	62.11	-	62.23	62.26	-
(444)	63.62	63.62	63.41	63.57	63.67	63.05
(534)	65.06	65.05	65.04	65.19	65.17	65.15
(642)	69.35	69.39	69.29	69.49	69.64	68.98

**Table 2:** The values of the direct energy band gap of In_2_O_3_ were determined using the Kubelka–Munk transformation.

Diffraction Parameters	In_2_O_3_ in the Absence of Surfactant			In_2_O_3_ in the Presence of Surfactant		
	550 °C	750 °C	900 °C	550 °C	750 °C	900 °C
E_g_ [eV]	3.28	3.41	3.51	3.63	3.87	4.33

## Data Availability

The original contributions presented in the study are included in the article; further inquiries can be directed to the corresponding authors.

## Abbreviations

The following abbreviations are used in this manuscript:

IPA	Isopropyl alcohol
DWA	Deionized water
SDS	Sodium dodecyl sulfate
NPs	Nanoparticles
RTA	Rapid thermal annealing
FTIR	Fourier transform infrared spectroscopy
XRD	X-ray diffraction
SEM	Scanning electron microscopy
DRS	Diffuse reflectance spectroscopy
CA	Contact angle

## References

[1] Goh K.W., Johan M., Wong Y.H. (2018). Enhanced structural properties of In_2_O_3_ nanoparticles at lower calcination temperature synthesized by co-precipitation method. Micro Nano Lett..

[2] Duua A.M., Mulsim F.J. (2018). Studying the Effect of Annealing Temperature on some Physical Properties of In_2_O_3_ Thin Films. Eng. Technol. J..

[3] Keles G., Sifa Ataman E., Taskin S.B., Polatoglu I., Kurbanoglu S. (2024). Nanostructured Metal Oxide-Based Electrochemical Biosensors in Medical Diagnosis. Biosensors.

[4] Savarimuthu E., Lalithambika K.L., Moses Ezhil Raj A., Nehru L.C., Ramamurth S., Thayumanavan A., Sanjeeviraja C., Jayachandranf M. (2007). Synthesis and materials properties of transparent conducting In_2_O_3_ films prepared by sol–gel-spin coating technique. J. Phys. Chem. Solids.

[5] Bagheri-Mohagheghi M.M., Shahtahmasebi N., Mozafari E., Shokooh-Saremi M. (2009). Effect of the synthesis route on the structural properties and shape of the indium oxide (In_2_O_3_) nano-particles. Physica.

[6] Rashed T.R. (2017). Theoretical Structures Study of Indium Oxide (In_2_O_3_) Cluster Using DFT Calculations. Asian J. Mater. Chem..

[7] Shah S., Hussain S., Ud Din S.T., Shahid A., Amu-Darko J.N., Wang M., Tianyan Y., Liu G., Qiao G. (2004). A review on In_2_O_3_ nanostructures for gas sensing applications. J. Environ. Chem. Eng..

[8] Yap B.K., Zhang Z., Thien G.S.H., Chan K.Y. (2023). Recent advances of In_2_O_3_-based thin-film transistors: A review. Appl. Surf. Sci..

[9] Simeonov S., Szekeres A., Spassov D., Anastasescu M., Stanculescu I., Nicolescu M., Aperathitis E., Modreanu M., Gartner M. (2022). Investigation of the Effects of Rapid Thermal Annealing on the Electron Transport Mechanism in Nitrogen-Doped ZnO Thin Films Grown by RF Magnetron Sputtering. Nanomaterials.

[10] Ullah H., Yamani Z.H., Qurashi A., Iqbal J., Safeen K. (2020). Study of the optical and gas sensing properties of In_2_O_3_ nanoparticles synthesized by rapid sonochemical method. J. Mater. Sci. Mater. Electron..

[11] Temiz M., Yildirim R.G., Bedir M.B., Öztaş M. (2020). Effect of boric acid concentrations on the characterization of the In_2_O_3_ thin films growth by spraying pyrolysis method. Dig. J. Nanomater. Biostruct..

[12] Feng C., Liu X., Wen S., An Y. (2019). Controlled growth and characterization of In_2_O_3_ nanowires by chemical vapor deposition. Vacuum.

[13] Faisal A.D., Aljubouri A.A., Khalef W.K. (2020). Synthesis of indium oxide nanowires on quartz substrate for gas sensor. J. Appl. Eng. Sci..

[14] Cho S. (2012). Effects of rapid thermal annealing on the properties of In_2_O_3_ thin films grown on glass substrate by RF reactive magnetron sputtering. Microelectron. Eng..

[15] Yan Y., Zhang Y., Zeng H., Zhang J., Cao X., Zhang L. (2007). Tunable synthesis of In_2_O_3_ nanowires, nanoarrows and nanorods. Nanotechnology.

[16] Attaf A., Bouhdjar A., Saidi H., Benkhetta Y., Bendjedidi H., Nouadji M., Lehraki N. (2015). Influence of Growth Time on Crystalline Structure, Morphologic and Optical Properties of In_2_O_3_ Thin Films. AIP Conf. Proc..

[17] Du X., Yu J., Xiu X., Sun Q., Tang W., Man B. (2019). Epitaxial growth and characterization of high quality In_2_O_3_ films on a-plane sapphire substrates by MOCVD. Vacuum.

[18] Kaleemulla S., Sivasankar Reddy A., Uthanna S., Sreedhara Reddy P. (2009). Physical properties of In_2_O_3_ thin films prepared at various oxygen partial pressures. J. Alloys Compd..

[19] Veeraswamy Y., Vijayakumar Y., Ramana Reddy M.V. (2014). Effect of Substrate on Structural and Optical Properties of In_2_O_3_ Thin Films Prepared by Electron Beam Evaporation. Asian J. Appl. Sci..

[20] Roopa, Pradhan B.K., Mauraya A.K., Chatterjee K., Pal P., Muthusamy S.K. (2024). High-sensitive and fast-responsive In_2_O_3_ thin film sensors for dual detection of NO_2_ and H_2_S gases at room temperature. Appl. Surf. Sci..

[21] He Y.Y., Zhao X., Cao Y., Zou X.X., Li G.D. (2016). Facile synthesis of In_2_O_3_ nanospheres with excellent sensitivity to trace explosive nitro-compounds. Sens. Actuators B.

[22] Xu H., Zhou Y., Zhang H., Dai M., Wang G., Yang G., Zhu Y. (2026). Glucose-assisted synthesis of In_2_O_3_ nanorods for high-performance ozone detection. Nanomaterials.

[23] Chen C., Moir J., Soheilnia N., Mahler B., Hoch L., Liao K., Hoepfner V., O’Brien P., Qian C., He L. (2015). Morphology-controlled In_2_O_3_ nanostructures enhance the performance of photoelectrochemical water oxidation. Nanoscale.

[24] Kim J., Rim Y.S., Chen H., Cao H.H., Nakatsuka N., Hinton H.L., Zhao C., Andrews A.M., Yang Y., Weiss P.S. (2015). fabrication of high-performance ultrathin In_2_O_3_ film field-effect transistors and biosensors using chemical lift-off lithography. ACS Nano.

[25] Liu Q., Liu Y., Wu F., Cao X., Li Z., Alharbi M., Abbas A.N., Amer M.R., Zhou C. (2018). Highly sensitive and wearable In_2_O_3_ nanoribbon transistor biosensors with integrated on-chip gate for glucose monitoring in body fluids. ACS Nano.

[26] Zhao Z., Mallon K., Chen M., Cui D., Tian F., AlBawardi S., Alsaggaf S., Amer M.R., Watson M.A., White M.A. (2025). In_2_O_3_ nanoribbon-based field-effect transistor biosensors for ultrasensitive detection of exosomal circulating microrna with peptide nucleic acid probes. ACS Nano.

[27] Kanchana Latha C., Raghasudha M., Aparna Y., Ramchander M., Ravinderd D., Jaipal K., Veerasomaiah P., Shridharf D. (2017). Effect of Capping Agent on the Morphology, Size and Optical Properties of In_2_O_3_ Nanoparticles. Mater. Res..

[28] Rashed T.R., Sariya D.A.A., Sahar Z.T. (2014). Preparation and Study of Indium Oxide Nanoparticles. Iraqi J. Appl. Phys..

[29] Kim J.M., Park J.K., Kim K.N., Kim C.H., Jang H.G. (2006). Synthesis of In_2_O_3_ nano-materials with various shapes. Curr. Appl. Phys..

[30] Chen C., Wei Y., Chen D., Jiao X. (2011). Indium oxide nanocrystals: Capping-agent-free synthesis, size-control mechanism, and high gas-sensing performance. Mater. Chem. Phys..

[31] Tao X., Sun L., Li Z., Zhao Y. (2010). Side-by-Side In(OH)_3_ and In_2_O_3_ Nanotubes: Synthesis and Optical Properties. Nanoscale Res. Lett..

[32] Yahia A., Attaf A., Saidi H., Dahnoun M., Khelifi C., Bouhdjer A., Saadi A., Ezzaouia H. (2019). Structural, optical, morphological and electrical properties of indium oxide thin films prepared by sol gel spin coating process. Surf. Interfaces.

[33] Vevdokymenko V.Y., Dobrozhan O., Pshenychnyi R., Opanasyuk A., Gnatenko Y., Bukivskii A., Bukivskij P., Gamernyk R., Кlymov O., Munoz-Sanjose V. (2023). The effect of annealing treatment on the structural and optical properties of nanostructured Cu_x_O films obtained by 3D printing. Mater. Sci. Semicond. Process..

[34] Bouhdjer A., Attaf A., Saidi H., Benkhetta Y., Aida M.S., Bouhaf I., Rhil A. (2016). Influence of annealing temperature on In_2_O_3_ properties grown by an ultrasonic spray CVD process. Optik.

[35] Huang P., Yahng H. (2018). A Design Method to Improve Temperature Uniformity on Wafer for Rapid Thermal Processing. Electronics.

[36] Trinidad-Urbina R.E., Castanedo-Pérez R., Torres-Delgado G., Sánchez-Martínez A., Ramírez-Bon R. (2004). Effects of Rapid Heat Treatments on the Properties of Cu_2_O Thin Films Deposited at Room Temperature Using an Ammonia-Free SILAR Technique. J. Electron. Mater..

[37] Usha K.S., Lee S.Y. (2004). Rapid thermal annealing treatment on WO_3_ thin films for energy efficient smart windows. Ceram. Int..

[38] Dogar S., Kim S.D. (2016). Effects of high-temperature rapid thermal annealing for seed layers on the crystallographic evolution in hydrothermal ZnO nanostructures. Mater. Sci. Semicond. Process..

[39] Martínez-Saucedo G., Castanedo-Pérez R., Torres-Delgado G., Mendoza-Galván A., Zelaya Ángel O. (2017). Cuprous oxide thin films obtained by dip-coating method using rapid thermal annealing treatments. Mater. Sci. Semicond. Process..

[40] Zhang X., Li Z., Fan J. (2022). An effect of rapid post-annealing temperature on the properties of cupric oxide thin films deposited by a remote plasma sputtering technique. Mater. Sci. Semicond. Process..

[41] Park C.J., Kim Y.W., Cho Y.J., Bobade S.M., Choi D.K. (2009). The Effects of Rapid Thermal Annealing on the Performance of ZnO Thin-Film Transistors. J. Korean Phys. Soc..

[42] Prepelita P., Stavarache I., Craciun D., Garoi F., Negrila C., Sbarcea B.N., Craciun V. (2019). Rapid thermal annealing for high-quality ITO thin films deposited by radio-frequency magnetron sputtering. Beilstein J. Nanotechnol..

[43] Kil S., Jeong J. (2023). Rapid thermal annealing effect on performance variations of solution processed indium–gallium–zinc-oxide thin-film transistors. AIP Adv..

[44] Tao J., Jiang X., Fan A., Hu X., Wang P., Dong Z., Wu Y. (2025). Effect of Rapid Thermal Annealing on the Characteristics of Micro Zn-Doped Ga_2_O_3_ Films by Using Mixed Atomic Layer Deposition. Nanomaterials.

[45] Matei A., Brîncoveanu O., Romaniţan C., Pachiu C., Ţucureanu V. (2025). Impact of Rapid Thermal Annealing Under Various Temperatures on the Yttrium Oxide Nanoparticles. Ann. West Univ. Timisoara Phys..

[46] Shen C., Xu N., Guan R., Zhang W. (2021). Highly sensitive ethanol gas sensor based on In_2_O_3_ spheres. Ionics.

[47] Joseph Panneerdoss I., Johnson Jeyakumar S., Ramalingam S., Jothibas M. (2015). Characterization of prepared In_2_O_3_ thin films: The FT-IR, FT-Raman, UV–Visible investigation and optical analysis. Spectrochim. Acta Part A Mol. Biomol. Spectrosc..

[48] Ayeshamariam A., Bououdina M., Sanjeeviraja C. (2013). Optical, electrical and sensing properties of In_2_O_3_ nanoparticles. Mater. Sci. Semicond. Process..

[49] Kelgenbaeva Z., Kelgenbaeva J.I., Ihara H., Omurzak E., Sulaimankulova S., Mashimo T. (2018). Thermal and Optical Properties of In and In_2_O_3_ Nanoparticles Synthesized Using Pulsed Plasma in Water. Phys. Status Solidi (A) Appl. Mater. Sci..

[50] Nistor M., Seiler W., Hebert C., Matei E., Perrière J. (2014). Effects of substrate and ambient gas on epitaxial growth indium oxidethin films. Appl. Surf. Sci..

[51] Trung H.M., Thien N.D., Lien D.T., Long N.N., Vu L.V. (2011). Synthesis and characterization of indium nanoparticles. VNU J. Sci. Math. Phys..

[52] Pecharsky V.K., Zavalij P.Y. (2009). Fundamentals of Powder Diffraction and Structural Characterisation of Materials.

[53] Rietveld H.M. (1969). A profile refinement method for nuclear and magnetic structures. J. Appl. Crystallogr..

[54] Romanitan C., Tudose I.V., Mouratis K., Popescu M., Pachiu C., Couris S., Koudoumas E., Suchea M.P. (2022). Structural investigations in Electrochromic Vanadium Pentoxide Thin Films. Phys. Status Solidi.

[55] Farva U., Lee H.W., Kim R.N., Lee D.G., Kang D.W., Kim J. (2021). Growth Temperature Influence on Atomic-Layer-Deposited In_2_O_3_ Thin Films and Their Application in Inorganic Perovskite Solar Cells. Nanomaterials.

[56] Khatibani A.B., Rozati S.M., Bargbidi Z. (2012). Preparation, Study and Nanoscale Growth of Indium Oxide Thin Films. Acta Phys. Pol. A.

[57] Saroni A., Alizadeh M., Rahman S.A., Meevasana W., Goh B.T. (2020). In-situ synthesis of In_2_O_3_-based heterojunction thin films for enhanced visible light photoelectrochemical performance. J. Power Sources.

[58] Matei A., Tucureanu V., Popescu M.C., Romanitan C., Mihalache I. (2019). Influence of Cu dopant on the morpho-structural and optical properties ZnO nanoparticles. Ceram. Int..

[59] Anand K., Kumar P., Thangaraj R. (2011). Effect of surfactant type on the microstructure and optical properties of In_2_O_3_ nanoparticles. J. Optoelectron. Adv. Mater..

[60] Dong H., Liu Y., Li G., Wang X., Xu D., Chen Z., Zhang T., Wang J., Zhang L. (2013). Hierarchically rosette-like In_2_O_3_ microspheres for volatile organic compounds gas sensors. Sensor Actuat. B Chem..

[61] Walter T. (2022). Measuring Non-Destructively the Total Indium Content and Its Lateral Distribution in Very Thin Single Layers or Quantum Dots Deposited onto Gallium Arsenide Substrates Using Energy-Dispersive X-ray Spectroscopy in a Scanning Electron Microscope. Nanomaterials.

[62] Hussain S.A., Al-Hilo E.A., Hatem A.H. (2016). Influence of Annealing on the Structural and Optical Characteristics of Indium Oxide Thin Films Prepared using Thermal Evaporation in a Vacuum Method. J. Adv. Phys..

[63] Nasriddinov A., Tokarev S., Fedorova O., Bozhev I., Rumyantseva M. (2022). In_2_O_3_ Based Hybrid Materials: Interplay between Microstructure, Photoelectrical and Light Activated NO_2_ Sensor Properties. Chemosensors.

[64] Shinde D.V., Ahn D.Y., Jadhav V.V., Lee D.Y., Shrestha N., Lee J.K., Lee H.Y., Mane R.S., Han S.H. (2014). A coordination chemistry approach for shape-controlled synthesis of indium oxide nanostructures and their photoelectrochemical properties. J. Mater. Chem. A.

[65] Sen S.K., Mortuza A.A., Manir M.S., Pervez M.F., Hossain S.M., Alam M.S., Haque M.A.S., Matin M.A., Hakim M.A., Huda A. (2020). Structural and optical properties of sol-gel synthesized h-MoO_3_ nanorods treated by gamma radiation. Nano Express.

[66] Souli M., Ajili L., Alhalaili B., Khadaraoui A., Vidu R., Kamoun-Turki N. (2021). Enhancement in structural, optical and morphological properties of sprayed In_2_O_3_ thin films induced by low energy electron beam irradiation. Mater. Sci. Semicond. Process..

[67] Sonsupap S., Swatsitang E., Maensiri S., Wongsaprom K. (2015). Synthesis and Characterization of Indium Oxide Nanoparticles Using Indium Nitrate and Polyvinylpyrrolidone (PVP) as Precursors. Chiang Mai J. Sci..

[68] Goswami S., Sharma A.K. (2019). Investigation of the optical behavior of indium oxide thin films with the aid of spectroscopic ellipsometry technique. Appl. Surf. Sci..

[69] Siciliano T., Di Giulio M., Tepore M., Genga A., Micocci G., Tepore A. (2012). In_2_O_3_ films prepared by thermal oxidation of amorphous InSe thin films. Thin Solid Films.

[70] Matei A., Stoian M., Brincoveanu O., Tucureanu V. (2023). Preparation and characterization of nanocomposites based on chitosan with ZnO-Curcumin. Ceram. Int..

[71] Méndez-López A., Zelaya-Ángel O., Toledano-Ayala M., Torres-Pacheco I., Pérez-Robles J.F., Acosta-Silva Y.J. (2020). The Influence of Annealing Temperature on the Structural and Optical Properties of ZrO_2_ Thin Films and How Affects the Hydrophilicity. Crystals.

[72] Krainer S., Hirn U. (2021). Contact angle measurement on porous substrates: Effect of liquid absorption and drop size. Colloid Surf. A-Physicochem. Eng. Asp..

[73] Son J.W., Fan L.W. (2021). Temperature dependence of the contact angle of water: A review of research progress, theoretical understanding, and implications for boiling heat transfer. Adv. Colloid Interface Sci..

[74] Geistlinger H., Zulfiqar B., Schlueter S., Amro M. (2021). New structural percolation transition in fractional wet 3D-porous media: A comparative μ-CT study. Water Resour. Res..

